# Patient-Centered Outcomes From Multiparametric MRI and MRI-Guided Biopsy for Prostate Cancer: A Systematic Review

**DOI:** 10.1016/j.jacr.2019.08.031

**Published:** 2020-04

**Authors:** Samuel W.D. Merriel, Victoria Hardy, Matthew J. Thompson, Fiona M. Walter, Willie Hamilton

**Affiliations:** a College of Medicine & Health, University of Exeter, Exeter, UK; b Department of Public Health & Primary Care, University of Cambridge, Cambridge, UK; c Department of Family Medicine, University of Washington, Seattle, Washington

**Keywords:** mpMRI, MRI biopsy, patient-centered outcomes, prostate cancer

## Abstract

**Objective:**

To identify and characterize patient-centered outcomes (PCOs) relating to multiparametric MRI (mpMRI) and MRI-guided biopsy as diagnostic tests for possible prostate cancer.

**Methods:**

Medline via OVID, EMBASE, PsycInfo, and the Cochrane Central register of Controlled Trials (CENTRAL) were searched for relevant articles. Hand searching of reference lists and snowballing techniques were performed. Studies of mpMRI and MRI-guided biopsy that measured any PCO were included. There were no restrictions placed on year of publication, language, or country for study inclusion. All database search hits were screened independently by two reviewers, and data were extracted using a standardized form.

**Results:**

Overall, 2,762 database search hits were screened based on title and abstract. Of these, 222 full-text articles were assessed, and 10 studies met the inclusion criteria. There were 2,192 participants featured in the included studies, all of which were conducted in high-income countries. Nineteen different PCOs were measured, with a median of four PCOs per study (range 1-11). Urethral bleeding, pain, and urinary tract infection were the most common outcomes measured. In the four studies that compared mpMRI or MRI-guided biopsy to transrectal ultrasound biopsy, most adverse outcomes occurred less frequently in MRI-related tests. These four studies were assessed as having a low risk of bias.

**Discussion:**

PCOs measured in studies of mpMRI or MRI-guided biopsy thus far have mostly been physical outcomes, with some evidence that MRI tests are associated with less frequent adverse outcomes compared with transrectal ultrasound biopsy. There was very little evidence for the effect of mpMRI and MRI-guided biopsy on emotional, cognitive, social, or behavioral outcomes.

Credits awarded for this enduring activity are designated “SA-CME” by the American Board of Radiology (ABR) and qualify toward fulfilling requirements for Maintenance of Certification (MOC) Part II: Lifelong Learning and Self-assessment. To access the SA-CME activity visit https://cortex.acr.org/Presenters/CaseScript/CaseView?CDId=OYPksTZKFeY%3d. SA-CME credit for this article expires December 27, 2022.

## Introduction

The current diagnostic tests for prostate cancer have important limitations, which can impact patients. Prostate biopsy via the transrectal (TRUS) or transperineal route under ultrasound guidance carry a risk of adverse effects [1], and both have a significant false-negative rate leading to potential underdiagnosis [2]. In recent years there has been increasing interest in the potential utility of multiparametric MRI (mpMRI) as a new diagnostic test for prostate cancer. mpMRI could avoid the need for up to 28% of men to undergo a prostate biopsy for possible prostate cancer if used as a prebiopsy triage test [3]. MRI-guided biopsy has been shown to increase the diagnostic accuracy for clinically significant prostate cancer and reduce the numbers of patients diagnosed with clinically insignificant prostate cancer [4,5].

Mortality benefits, diagnostic accuracy, and adverse effects are all important clinical outcomes of diagnostic tests, but they are not the only elements that need to be considered. The Agency for Healthcare Research and Quality Effective Healthcare Program White Paper series on diagnostic test evaluation proposed that, in addition to the clinical outcome, a medical test can have emotional, social, cognitive, and behavioral outcomes for patients. These outcomes can be positive or negative, and they are not restricted to the medical test itself, but the entire diagnostic pathway [6].

Outcomes that are considered to have most importance or meaning to patients are often referred to as patient-centered outcomes (PCOs), although a precise definition of PCOs has not yet been reached [7]. The Patient-Centered Outcomes Research Institute (PCORI) has been established to support and conduct research into the comparative effectiveness of health care interventions to inform patient and clinical decision making. PCOs have three domains [8]:

1. Assessment of harms and benefits to inform decision making, highlighting comparisons and outcomes that matter to people
2. A focus on outcomes that people notice and care about
3. The incorporation of a wide variety of settings and diversity of participants

Among the PCORI portfolio, there is some ongoing work exploring the most important outcomes for patients from diagnostic tests [9].

This systematic review aims to summarize and compare the current evidence relating to PCOs for mpMRI or MRI-guided biopsy as a diagnostic test in men suspected of having prostate cancer.

## Methods

The protocol for this systematic review has been published on PROSPERO [10].

In summary, databases including Medline via OVID, EMBASE, PsycInfo, and the Cochrane Central register of Controlled Trials (CENTRAL) were selected to search for relevant articles. The Cochrane Collaboration recommends combining the test(s) of interest with the specific condition to refine searches [11]. This approach was merged with pretested search filters developed by the Scottish Intercollegiate Guidelines Network [12] for “diagnostic studies” and “patient issues” to achieve balance between the sensitivity and precision of the search strategy (see e-only Appendix 1). Hand searching and snowballing techniques from reference lists of systematic reviews and key references were performed to identify potentially relevant studies not captured by database searches.

The inclusion criteria were: (1) studies of MRI-guided biopsy or mpMRI for possible prostate cancer diagnosis and (2) PCOs included as an outcome measure in the study (as primary or secondary outcomes).

There were no limits set on date of publication, language, or study design. All database search hits were assessed independently against the inclusion criteria by two reviewers (S.M., V.H.). Disagreements were resolved with a third reviewer (W.H.). Full-text articles were reviewed, and data were extracted from full-text studies using a standardized form piloted in three studies and iteratively developed to capture all possible PCOs. Study quality for randomized controlled trials (RCTs) was assessed with the Cochrane Risk of Bias tool [13], and the MINORS checklist [14] was used for nonrandomized studies. A narrative approach was used to synthesize findings due to significant study heterogeneity. This manuscript was written following the Preferred Reporting Items for Systematic Reviews and Meta-Analyses statement [15] (see e-only Appendix 2).

## Results

**Fig 1:**
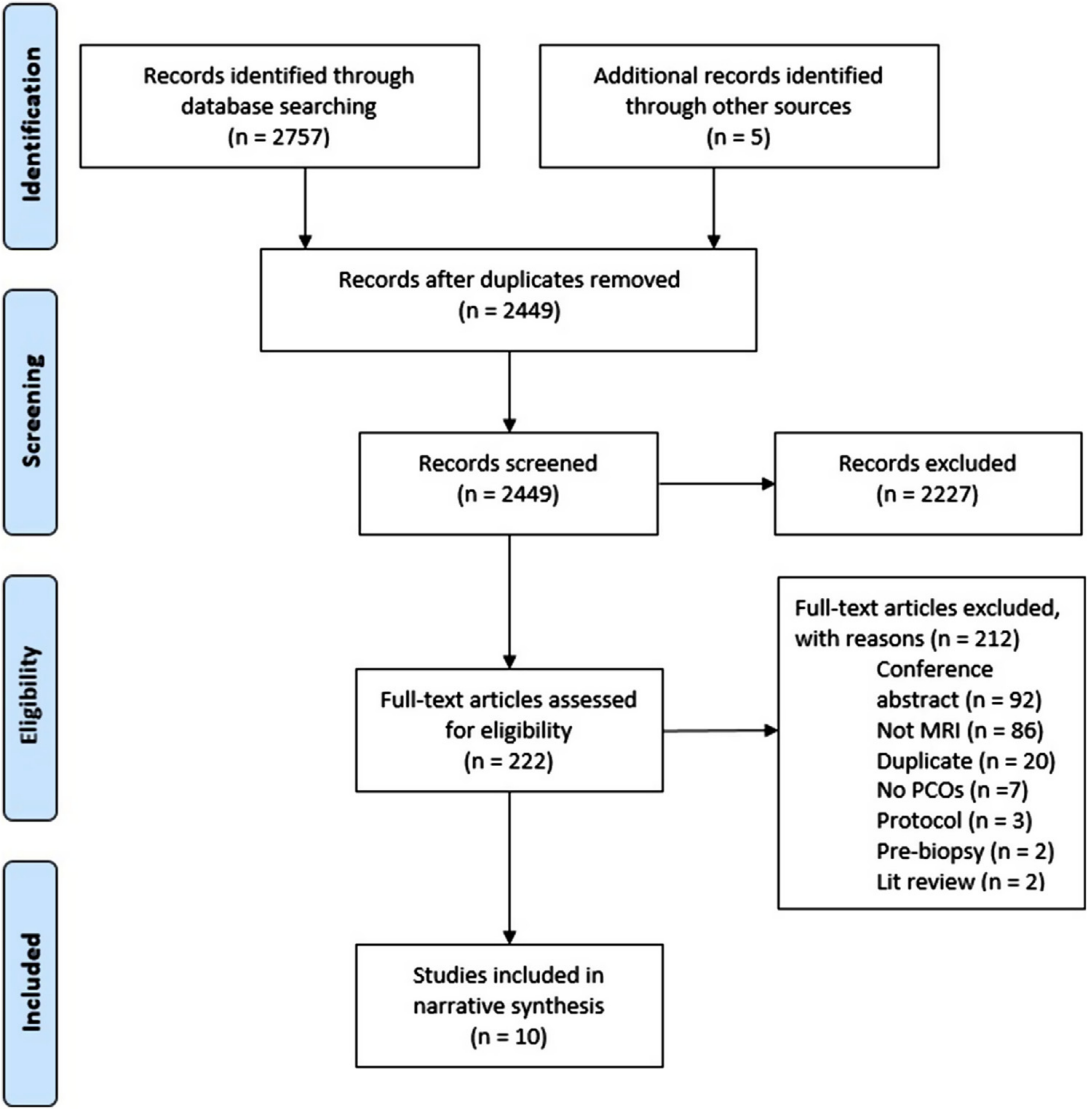
Preferred Reporting Items for Systematic Reviews and Meta-Analyses flow diagram (from Moher et al [37]).

In all, 2,762 records were identified through database and hand searching. After removal of duplicates and screening of titles and abstracts, 222 full-text articles were assessed. Ten publications were included in the systematic review. A full breakdown of study selection and reasons for full-text exclusions is in Figure 1.

### Study Characteristics

Seven of the included studies were performed in European countries, two in the United States and one in Australia. Mean ages for participants in included studies ranged from 63 to 66 years, and the numbers of participants ranged from 8 to 576. Studies varied widely in terms of design, participant numbers, and outcomes measured. Table 1 contains full details of all included studies. Of the 10 included studies, 4 [5,^16^, ^17^, ^18^ were assessed as having a low risk of bias (see Table 2).

**Table 1:** Details of included studies

Author (Year) [Reference]	Country	Study Design	Participants	Mean Age (y)	Diagnostic Test(s)	Follow-up (days)	Outcomes Measured
Ahmed et al (2017) [16]	UK	Prospective cohort	576	63.4	mpMRI, MRI-GB, and TRUS-GB in same patient	30	Physical
Egbers et al (2015) [17]	Germany	Cross-sectional	54	68 (median)	MRI-GB after negative TRUS-GB	7	Physical
Hadaschik et al (2011) [24]	Germany	Prospective	106	66	mpMRI and fusion MRI–TRUS biopsy	1	Physical
Kasivisvanathan et al (2018) [5]	Multiple	RCT	500	64.4	mpMRI ± MRI-GB or TRUS-GB	30	Physical, QoL
Kuru et al (2013) [21]	Germany	Prospective	347	65	Fusion MRI–TRUS biopsy	28	Physical
Miah et al (2018) [20]	UK	Prospective	249	63.7	mpMRI and TTPM	56	Physical, QoL
Pokorny et al (2014) [18]	Australia	Prospective	223	63	mpMRI, MRI-GB and TRUS-GB in same patient		Physical
Powell et al (2014) [23]	USA	Prospective	30	Unreported	mpMRI with colorectal or prostatic coil	0	Physical
Stanley et al (2016) [28]	Ireland	Case-control	8	49 (median)	MRI		Emotional, QoL
Tilak et al (2015) [22]	USA	Retrospective-prospective	99	66.01	Manual or robotic MRI-guided TTPM		Physical

mpMRI = multiparametric MRI; MRI-GB = MRI-guided biopsy; QoL = quality of life; RCT = randomized controlled trial; TRUS = transrectal ultrasound biopsy; TRUS-GB = TRUS-guided biopsy; TTPM = transperineal temple prostate mapping biopsy.

**Table 2:** Study quality assessment

Author (Year) [Reference]	Randomization	Deviation	Missing Data	Measurement	Selection	Overall
Randomized studies∗						
Kasivisvanathan et al (2018) [5]	Low risk	Low risk	Low risk	Low risk	Low risk	Low risk
Powell et al (2014) [23]	Low risk	Medium risk	Low risk	High risk	Low risk	Some concerns

Author (Year) [Reference]	Aim	Consecutive Pts	Prospective Data	End-points	Unbiased Assess	Fup Appropriate	Loss to Fup	Size Calc	Subtotal	Adequate Control	Contem-porary	Equal Groups	Analysis	Total
Nonrandomized studies†														
Ahmed et al (2017) [16]	2	2	2	2	2	0	2	2	14‡					
Egbers et al (2015) [17]	2	2	2	2	2	1	2	0	13‡					
Hadaschik et al (2011 [24])	2	1	0	2	0	0	0	0	5§					
Kuru et al (2013) [21]	2	1	1	1	1	1	0	0	7‖					
Miah et al (2018) [20]	1	2	2	2	2	1	2	0	12‖					
Pokorny et al (2014) [18]	2	2	2	2	1	1	2	0	12‖	2	2	2	2	20‡
Stanley et al (2016) [28]	2	1	2	1	0	1	0	0	7‖	1	2	1	1	12‖
Tilak et al (2015) [22]	2	1	1	1	0	1	0	0	6§	1	1	1	2	11‖

Calc = calculation; Fup = follow-up; Pts = patients.

∗ Risk of bias assessment for included randomized controlled trials [13].

† Study quality assessment of nonrandomized studies (two reported and adequate; one reported, not adequate; zero not reported; subtotal of 16; total of 24.

‡ High quality [14].

§ Low quality.

‖ Medium quality.

Nineteen different outcomes were measured across the 10 included studies, measuring an average of 4.9 outcomes per study. The number of outcomes measured in individual studies varied from 1 to 11. Included publications very seldom justified the selection of outcomes measured (see Table 3 for further information on outcomes measured).

**Table 3:** PCOs from all included studies

PCO (Measure)	Studies of mpMRI or MRI-Guided Biopsy and TRUS				Studies of mpMRI or MRI-Guided Biopsy Only					
	Ahmed et al (2017) [16]	Egbers et al (2015) [17]	Kasivisvanathan et al (2018) [5]	Pokorny et al (2014) [18]	Hadaschik et al (2011) [24]	Kuru et al (2013) [21]	Miah et al (2018) [20]	Powell et al (2014) [23]	Stanley et al (2016) [28]	Tilak et al (2015) [22]
Physical outcomes										
Pain	64%	2 of 10 (VAS)	1 of 10 (VAS)				61.8%	2.7 of 10 (VAS)		
Dysuria	46%									
Urethral bleeding	67%	51%	30.2%	0		50.6%	88.4%			5.92%
Hemato- spermia	55%	36%	32.1%							
Rectal bleeding		16%	14.2%							
Hematoma					0.94% (unreported)	13%	54.6%			16.07%
Acute urinary retention	10%		1.4%		1.9% (unreported)		22.55%			
UTI	6%		5.4%		0 (unreported)	1%	9.2%			
Fever		2.2%	4.2%							
Sepsis	1%		0.4% (notes review)	0						
Erectile dysfunction	14%		10.8%			26.3%	9.02 (IIEF)			
LUTS							0.83 (IPSS)			
Urinary incontinence			6.1%							
Vasovagal				0.45%						
Quality of life outcomes										
Quality of life			−0.004 (EQ5-D)				0.19 (IPSS QoL)			
Satisfaction									1.67 (4-point Likert scale)	
Emotional outcomes										
Anxiety									39%	

Outcomes were measured through self-report unless otherwise stated, and were presented as proportions who reported the outcome. EQ5-D = EuroQol-5 Dimensions; IIEF = International Index of Erectile Function; IPSS = International Prostate Symptom Score; ISS = International Prostate Symptom Score; LUTS = lower urinary tract symptoms; mpMRI = multiparametric MRI; PCO = patient-centered outcome; QoL = quality of life; TRUS = transrectal ultrasound; Unreported =– authors did not report outcome measure; UTI = urinary tract infection; VAS = visual analogue scale.

### Physical Effects

#### Bleeding

Bleeding after investigation was the most commonly measured outcome. Bleeding was categorized as urethral bleeding, rectal bleeding, hematospermia, or hematoma. Bleeding was measured through self-report from patients via survey or interview in all studies (one unreported), and reporting occurred between 7 and 56 days after biopsy. The proportion of patients reporting some type of bleeding after biopsy varied between studies from 0% to 88.4% [6,^16^,^17^,^19^, ^20^, ^21^, ^22^.

#### Pain

Pain was measured in five studies: three utilized a 10-point visual analogue scale [6,^17^,23], two relied on patient self-report [16,20], and one measured the presence of pain 4 days after biopsy [17]. Pain was measured between 0 and 56 days postprocedure. Kasivisvanathan et al found a mean visual analogue scale of 1 for MRI-guided biopsy and 2 for TRUS biopsy, though without performing significance testing [5]. Egbers et al found a significantly lower pain score from patients undergoing MRI-guided biopsy compared with TRUS biopsy (median visual analogue scale 2 versus 3, *P* < .005) [17].

#### Infection

Urinary tract infection and urosepsis are also important potential adverse effects from undergoing a prostate biopsy and were measured in five publications. A mixture of measures, including recorded fever, urine culture, clinical notes review, and patient self-report, were utilized to asses for signs of infection. Sepsis (0.4%-1.6%) [6,^16^,19] occurred less commonly than urinary tract infection (1%-9.2%) [6,^16^,^21^,^20^,24] across the individual studies, which measured this outcome.

#### Urinary Retention

Four studies [6,^16^,^20^,25] assessed whether patients went into acute urinary retention after undergoing a prostate biopsy, measured 30 to 56 hours after the biopsy. Consistent with most other outcomes, this was mostly measured by patient self-report. In the study by Miah et al [20], which used MRI-guided transperineal template mapping biopsy, 22.6% (56 of 249) of men suffered urinary retention, whereas it was much less common in the other studies that used TRUS-guided biopsy (1%-10%) or MRI-TRUS fusion biopsy (1.9%).

#### Erectile Dysfunction

Problems achieving or maintaining erection after prostate biopsy are recognized as a potential adverse effect [26]. One study measured this using the International Index of Erectile Function [20]; and three used self-report [5,^16^,27]. Follow-up time for this outcome was also longer than for others (median 30 days, range 28-56). Erectile dysfunction occurred in between 10.8% and 26.3% of men.

#### Lower Urinary Tract Symptoms

Symptoms such as waking frequently in the night to pass urine, passing urine often, and having a poor stream are among a group of symptoms commonly referred to as lower urinary tract symptoms (LUTS). LUTS usually occur due to diseases of the prostate or the bladder, though they can also occur after prostate biopsy. Miah et al measured LUTS using the International Prostate Symptoms Score [20], and showed a small increase in the presence of LUTS post-biopsy (10.93 ± 6.77 prebiopsy versus 11.76 ± 6.56 postbiopsy, *P* = .024).

### Emotional Effects

Stanley et al was the only study to specifically measure anxiety relating to undergoing an MRI scan and found that there was no difference whether patients received an intervention aimed at reducing anxiety or not. In both the intervention and control groups, 39% of participants reported preprocedure anxiety [28].

### Quality of Life

Kasivisvanathan et al assessed patients undergoing prostate biopsy for changes in quality of life using the EuroQol-5 Dimensions and demonstrated a nonsignificant difference after TRUS-guided biopsy (−0.27; 95% confidence interval [CI] −1.88 to 1.33) compared with MRI-guided biopsy (−0.0004; 95% CI −0.028 to 0.020) [5]. Miah et al measured quality of life using a subsection of the International Prostate Symptoms Score involving one question with a 7-point Likert scale (7 being low), showing a mean score of 1.76 (±1.39) postbiopsy [20].

### mpMRI/MRI Guided Biopsy Versus TRUS-Guided Biopsy

Four studies included mpMRI or MRI-guided biopsy and TRUS-guided biopsy. Kasivisvanathan et al randomized patients to mpMRI, with MRI-guided biopsy if a lesion was detected or TRUS-guided biopsy [5] for a multicenter, randomized, noninferiority trial in 11 countries. Two studies (one in the UK and one in Australia) compared mpMRI with subsequent MRI-guided biopsy to TRUS-guided biopsy in the same patients in prospective cohort studies [16,18] and one in a cross-sectional study in Germany [17]. Table 4 shows a comparison of the outcomes measured between MRI- and TRUS-guided biopsy.

**Table 4:** Adverse PCOs from studies comparing mpMRI and MRI-guided biopsy to TRUS-guided biopsy

Author (Year) [Reference]	Pain	Urethral Bleeding	Hematospermia	Rectal Bleeding	Urinary Retention	Fever	UTI	Urosepsis	ED	Incontinence	QoL	Vasovagal
Ahmed et al (2017) [16]	∗	‡	‡	‡	‡	‡	‡	‡	‡	‡	‡	‡
Egbers et al (2015) [17]	∗	∗	†	∗	‡	∗	‡	‡	‡	‡	‡	‡
Kasivisvanathan et al (2018) [5]	∗	∗	∗	∗	†	∗	†	∗	∗	†	∗	‡
Pokorny et al (2014) [18]	‡	∗	‡	‡	‡	‡	‡	∗	‡	‡	‡	†

ED = erectile dysfunction; mpMRI = multiparametric MRI; QoL = quality of life; TRUS = transrectal ultrasound; UTI = urinary tract infection.

∗ Less frequent from mpMRI or MRI-guided biopsy

† Less frequent from TRUS-guided biopsy.

‡ Patient-centered outcome not measured

## Discussion

### Key Findings

This systematic review of PCOs associated with mpMRI and MRI-guided biopsy for prostate cancer found wide variation in study quality, PCOs measured, tools used for measurement, follow-up of patients, and outcomes. In the four studies that compared mpMRI and subsequent MRI-guided biopsy with TRUS-guided biopsy, most adverse PCOs were less frequent with MRI testing. Pain and bleeding were the most commonly measured PCOs. In contrast, there were no published studies measuring any cognitive, social, or behavioral outcomes of mpMRI or MRI-guided biopsy. Meta-analysis was not possible due to significant heterogeneity.

### Comparison With Existing Literature

This is the first systematic review of PCOs associated with mpMRI and MRI-guided biopsy for prostate cancer, as far as the authors are aware. Glaser et al performed a literature review of the effects of prostate biopsy on urinary symptoms, erectile function, and anxiety after early reports in the field [26]. The authors looked at TRUS-guided biopsy only and considered the relationship of these outcomes with factors such as analgesic approaches and type of approach to TRUS-guided biopsy. They found evidence suggesting a transient increase in LUTS, and a relationship between TRUS-guided biopsy and erectile dysfunction in the short term. The authors considered that the impact on erectile dysfunction needed further research to determine the etiology of this effect. There was limited justification for choosing to focus on these outcomes, or why others were omitted.

Efficace et al undertook a systematic review of health-related quality of life measurements performed in RCTs relating to prostate carcinoma treatments [29]. The authors found a range of health-related quality of life assessments; however, some studies had methodological limitations that could have affected the measurement of health-related quality of life. The same authors assessed the methodological quality of patient-reported outcomes in RCTs with prostate cancer patients in 2014 [30]. The quality of patient-reported outcomes improved over time, and approximately 20% of the assessed patient-reported outcomes were deemed to collate sufficient detail to inform clinical practice and health policy. These two systematic reviews focused only on studies of conventional prostate cancer treatments, excluding any other interventions such as diagnostic testing or alternative therapies.

There is growing recognition of the importance of PCOs for diagnostic tests within radiology, especially in the United States, after the establishment of the PCORI [31]. There have been methodological challenges in identifying and measuring PCOs relating to diagnostic tests that are still being overcome. Many of the direct effects on patients from undergoing an imaging test are short term in nature, and not easily captured with existing measures used in research [32]. The relationship between these short-term effects and the ultimate patient outcome may be tenuous, because diagnostic testing makes up just one element in a patient’s illness journey [33].

This review found very little evidence of patient involvement in identifying outcomes to measure in studies of prostate cancer diagnostic tests. This finding is consistent with Mathers et al, who showed that, up until 2006, there was minimal patient engagement to determine the important patient outcomes for radiology research [34]. A recent study of outcomes in primary care for imaging tests interviewed patients who had undergone x-ray, CT scan, MRI, or ultrasound in the previous 12 months. The four key themes for outcomes that were identified from patients were knowledge gained from the test, test contribution to overall health care journey, physical experiences during the test, and impacts of the testing process on emotions [35]. Studies in this systematic review considered only the latter two patient priorities, but omitted the knowledge gained or the impact of MRI- or TRUS-guided biopsy on the overall patient journey.

### Strengths and Limitations

This study followed a systematic and comprehensive methodological approach to understand which PCOs have been measured in studies of diagnostic tests for prostate cancer. Published high-quality search strategies were adapted for the purposes of this study. The search strategy and definition used for PCOs were deliberately broad to identify as many relevant studies as possible to obtain a clear picture of all current research. Some recent studies comparing mpMRI and MRI-guided biopsy to TRUS-guided biopsy were obtained, allowing tentative conclusions to be drawn between the two diagnostic tests regarding their comparative effectiveness.

However, this systematic review has some important limitations affecting the generalizability of the results. PCO measures have not yet been clearly defined, and designing a systematic search strategy to capture all studies measuring PCOs was problematic. It is possible some studies that could have been included were missed despite our thorough search methodology. The included studies varied widely in a number of areas, making meta-analysis between PCOs for mpMRI or MRI-guided biopsy and TRUS-guided biopsy impossible. Most studies included in the study had at least some risk of bias based on the quality assessment, and there were few data reported on PCOs other than physical outcomes of undergoing testing.

### Implications for Policy and Practice

Within the limited evidence currently available, there is some indication that mpMRI and MRI-guided biopsy may perform better than TRUS-guided biopsy in terms of PCOs. TRUS-guided biopsy is the current standard diagnostic test for prostate cancer, despite its known limitations [1]. Following on from the PROMIS [16] and PRECISION [5] trials showing the higher diagnostic accuracy of mpMRI and MRI-guided biopsy for prostate cancer, the National Institute for Health and Care Excellence in the UK has recently updated guidelines for prostate cancer to include a recommendation for prebiopsy mpMRI in all patients with possible prostate cancer [36]. MRI-based diagnostic pathways for prostate cancer need further investigation to determine the best design and the economic impacts of these pathways. Integration of PCOs into this research would provide more robust evidence to determine whether mpMRI and MRI-guided biopsy truly do outperform TRUS-guided biopsy in key domains other than diagnostic accuracy.

## Take-Home Points

▪ Studies of mpMRI and MRI-guided biopsy for prostate cancer have mostly measured physical PCOs, with very limited evidence about the emotional, cognitive, behavioral, and social effects of testing.
▪ Some evidence suggests mpMRI and MRI biopsy are associated with fewer adverse PCOs compared with TRUS biopsy.
▪ There is no evidence of patient engagement or involvement in the selection of PCOs for studies of mpMRI and MRI biopsy for possible prostate cancer.

Credits awarded for this enduring activity are designated “SA-CME” by the American Board of Radiology (ABR) and qualify toward fulfilling requirements for Maintenance of Certification (MOC) Part II: Lifelong Learning and Self-assessment. To access the SA-CME activity visit https://cortex.acr.org/Presenters/CaseScript/CaseView?CDId=OYPksTZKFeY%3d. SA-CME credit for this article expires December 27, 2022.
