# Supplementary material for: Patient-Centered Outcomes From Multiparametric MRI and MRI-Guided Biopsy for Prostate Cancer: A Systematic Review
Source: J Am Coll Radiol. 2020 Apr;17(4):486–95. doi: 10.1016/j.jacr.2019.08.031 (PMC7132450; doi:10.1016/j.jacr.2019.08.031)
Supplement: Appendix 1 [file mmc1.docx]

Appendix 1 – Search strategies for individual databases

**Medline**

1. (prostat* adj3 (cancer* or carcinoma* or malignan* or tumo?r* or neoplas* or adeno*)).tw.
2. Exp Prostatic neoplasms/
3. Exp Prostatic Intraepithelial Neoplasia/
4. Exp prostate/
5. OR/1-4
6. exp "Sensitivity and Specificity"/
7. sensitivity.tw.
8. specificity.tw.
9. ((pre-test or pretest) adj probability).tw.
10. post-test probability.tw.
11. predictive value$.tw.
12. likelihood ratio$.tw.
13. OR/6-12
14. “multiparametric magnetic resonance imaging”.mp
15. mpMRI.mp
16. “magnetic resonance imaging”.mp
17. MRI.mp
18. OR/14-18
19. “prostate biopsy”
20. TRUS
21. Transrectal
22. 19 AND 21
23. Transperineal
24. 19 AND 23
25. Mapping.tw
26. 19 AND 25
27. Template.tw
28. 19 AND 27
29. 19 OR 20 OR 22 OR 24 OR 26 OR 28
30. “patient-focus*”.mp
31. “patient-centred”.mp
32. “patient-centered”.mp
33. “patient reported”.mp
34. PROM
35. PROMS
36. Patient outcome assessment/
37. “patient experience”.tw
38. “quality of life”.tw
39. QoL.tw
40. OR/30-39
41. 5 AND 13 AND 40
42. 5 AND 29 AND 40

**EMBASE**

1. (prostat* adj3 (cancer* or carcinoma* or malignan* or tumo?r* or neoplas* or adeno*)).tw.
2. Exp prostate/
3. Or/1-3
4. “multiparametric magnetic resonance imaging”
5. “multiparametric MRI”
6. mpMRI
7. “magnetic resonance imaging”
8. MRI
9. Or/4-9
10. “prostate biopsy”.tw
11. TRUS
12. Transrectal
13. 10 AND 12
14. Transperineal
15. 10 AND 14
16. Mapping
17. 10 AND 16
18. Template
19. 10 AND 18
20. 10 OR 11 OR 13 OR 15 OR 17 OR 19
21. “patient-focused”.tw
22. “patient-centred”.tw
23. “patient-centered”.tw
24. “patient reported outcome”
25. PROM
26. PROMS
27. Outcome assessment/
28. Health status indicator/
29. Outcomes research/
30. ((patient* or self or client* or subject* or participant* or lived or personal or consumer* or “service user” or “service users”) NEAR/3 (report* or relate* or view* or expectation* or perception* or perspective* or experience*or measure* or impact* or effect*)).tw
31. Or/21-29
32. 3 AND 9 AND 31
33. 3 AND 20 AND 31

**PSYCINFO**

1. Prostate.af
2. (prostat* adj3 (cancer* or carcinoma* or malignan* or tumo?r* or neoplas* or adeno*)).af
3. 1 or 2
4. mpMRI.af
5. mri.af
6. “multiparametric magnetic resonance imaging”.af
7. “multiparametric mri”.af
8. “magnetic resonance imaging”.af
9. 4 or 5 or 6 or 7 or 8
10. TRUS.af
11. “prostate biopsy”.af
12. Transrectal.af
13. Transperineal.af
14. Mapping.af
15. Template.af
16. 11 and 12
17. 11 and 13
18. 11 and 14
19. 11 and 15
20. 10 or 11 or 16 or 17 or 18 or 19
21. “quality of life”.af
22. “health related quality of life”.af
23. “patient outcome$”.af
24. “patient reported outcome$”.af
25. “patient centred”.af
26. “patient centered”.af
27. “patient centred outcome$”.af
28. “patient centered outcome$”.af
29. PRO.af
30. PROM.af
31. PROMs.af
32. Qol.af
33. Hrqol.af
34. Hrql.af
35. PREM.af
36. “patient experience”.af
37. 21 or 22 or 23 or 24 or 25 or 26 or 27 or 28 or 29 or 30 or 31 or 32 or 33 or 34 or 35 or 36
38. 3 and 9 and 37
39. 3 and 20 and 37

**CENTRAL**

#1 Prostat*(cancer or neoplasm* or carcin* or tumour* or tumor* or malignan* or neoplasia or adenocarcinoma*): ti,ab,kw

#2 MeSH descriptor: [Prostatic neoplasms] explode all trees

#3 MeSH descriptor: [Prostatic Intraepithelial Neoplasia] explode all trees

#4 MeSH descriptor: [Prostate] explode all trees

#5 #1 or #2 or #3 or #4

#6 “patient-centred”:ti,ab,kw

#7 “patient-centered”:ti,ab,kw

#8 PCO

#9 “patient-focused”:ti,ab

#10 PROMS

#11 “patient reported outcome*”

#12 ((patient* or self or client* or subject* or participant* or lived or personal or consumer* or “service user” or “service users”) NEAR/3 (report* or relate* or view* or expectation* or perception* or perspective* or experience*or measure* or impact* or effect*)):ti,ab

#13 MeSH descriptor: [Patient Reported Outcome Measures] explode all trees

#14 MeSH descriptor: [Patient Outcome Assessment] explode all trees

#15 #6 or #7 or #8 or #9 or #10 or #11 or #12 or #13 or #14

#16 “multiparametric magnetic resonance imaging”:ti,ab,kw

#17 “multiparametric MRI:ti,ab,kw

#18 mpMRI

#19 “magnetic resonance imaging”:ti,ab,kw

#20 MRI

#21 #16 or #17 or #18 or #19 or #20

#22 TRUS

#23 “prostate biopsy”:ti,ab,kw

#24 transrectal

#25 transperineal

#26 mapping

#27 template

#28 #23 and #24

#29 #23 and #25

#30 #23 and #26

#31 #23 and #27

#32 #22 or #23 or #28 or #29 or #30 or #31

#33 #5 and #15 and #21

#34 #5 and #15 and #32
